# Altered long‐ and short‐range functional connectivity density associated with poor sleep quality in patients with chronic insomnia disorder: A resting‐state fMRI study

**DOI:** 10.1002/brb3.1844

**Published:** 2020-09-16

**Authors:** Fuqing Zhou, Yanyan Zhu, Yujun Zhu, Muhua Huang, Jian Jiang, Laichang He, Suhua Huang, Xianjun Zeng, Honghan Gong

**Affiliations:** ^1^ Department of Radiology The First Affiliated Hospital Nanchang University Nanchang China; ^2^ Neuroimaging Laboratory Jiangxi Province Medical Imaging Research Institute Nanchang China; ^3^ Department of Radiology Jiangxi Province Children's Hospital Nanchang China; ^4^ Department of Respiratory The People’s Hospital of Yichun City Yichun China

**Keywords:** chronic insomnia disorder, functional connectivity density, esting‐state fMRI, sleep quality

## Abstract

**Introduction:**

Previous neuroimaging studies have suggested that brain functional impairment and hyperarousal occur during the daytime among patients with chronic insomnia disorder (CID); however, alterations to the brain's intrinsic functional architecture and their association with sleep quality have not yet been documented.

**Methods:**

In this study, our aim was to investigate the insomnia‐related alterations to the intrinsic connectome in patients with CID (*n* = 27) at resting state, with a data‐driven approach based on graph theory assessment and functional connectivity density (FCD), which can be interpreted as short‐range (intraregional) or long‐range (interregional) mapping.

**Results:**

Compared with healthy controls with good sleep, CID patients showed significantly decreased long‐range FCD in the dorsolateral prefrontal cortices and the putamen. These patients also showed decreased short‐range FCD in their multimodal‐processing regions, executive control network, and supplementary motor‐related areas. Furthermore, several regions showed increased short‐range FCD in patients with CID, implying hyper‐homogeneity of local activity.

**Conclusions:**

Together, these findings suggest that insufficient sleep during chronic insomnia widely affects cortical functional activities, including disrupted FCD and increased short‐range FCD, which is associated with poor sleep quality.

## Graphical abstract

Using graph theory assessment and functional connectivity density (FCD) methods to investigate the insomnia‐related alterations to the intrinsic connectome in patients with CID patients, we found that insufficient sleep during chronic insomnia widely affects cortical functional activities, including disrupted FCD and increased short‐range FCD, which is associated with poor sleep quality.

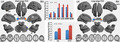

## 1 INTRODUCTION

Chronic insomnia disorder (CID) is characterized by difficulty initiating or maintaining sleep or by nonrestorative sleep that lasts for at least three months. CID is accompanied by significant distress or impairments in social, occupational, or other important functional areas (Ohayon & Roth, 2003; Spiegelhalder & Riemann, 2013). Although insomnia is a common health complaint, only a few structural and functional imaging studies are available regarding the pathophysiology of this disorder. Recently, regional cortical atrophy in the hippocampus, orbitofrontal cortex, and anterior cingulate cortex (ACC) (Altena, Vrenken, van der Werf, van den Heuvel, & van Someren, 2010; Joo et al., 2013; O’Byrne, Rosa, Gouin, & Dang‐Vu, 2014), together with reduced white matter integrity in the internal capsule, corona radiata, corpus callosum and thalamus (Li et al., 2016a; Spiegelhalder et al., 2014), and disrupted cortical structural connectivity (Hutton, Draganski, Ashburner, & Weiskopf, 2009), have been detected in patients with CID. Functional neuroimaging studies have also provided important evidence regarding insomnia‐related dysfunction (E. Altena et al., 2008; Fortier‐Brochu, Beaulieu‐Bonneau, Ivers, & Morin, 2012) and hyperarousal states (Drummond et al., 2013) in the brains of patients with CID.

Resting‐state functional magnetic resonance imaging (rs‐fMRI) is a relatively recent development technique that allows investigators to investigate insomnia‐related neural architecture to understand the neural mechanisms that underlie insomnia. Most of the brain's energy consumption is devoted to intrinsic activities, such as spontaneous blood oxygenation level‐dependent (BOLD) signaling, as observed on fMRI scans. A growing body of rs‐fMRI literature has revealed abnormal spontaneous neuronal activity and functional connectivity in people with neurological and psychiatric disorders. Consistent with such research, disrupted emotional circuit connectivity (Huang et al., 2012) and hyperconnectivity in sensory regions, motor regions (Killgore, Schwab, Kipman, DelDonno, & Weber, 2013; Li et al., 2014), and salience networks (Chen, Chang, Glover, & Gotlib, 2014) have been detected in recent rs‐fMRI studies of patients with insomnia. More recently, altered regional homogeneity (ReHo)(Dai et al., 2014; Wang et al., 2016) was related to emotional changes (i.e., depression). However, these studies have ignored the importance of the brain's intrinsic functional architecture in each voxel. Regions including the default‐mode network (DMN), which shows high functional connectivity density (FCD)(Tomasi & Volkow, 2010) and high rates of cerebral blood flow and glucose metabolism (Liang, Zou, He, & Yang, 2013), serve to integrate diverse information sources. Such regions with integrative functions might reflect the increased complexity of the connectome as a whole, which differs from the approaches of other intrinsic functional connectivity (iFC) studies, such as seed‐based approaches or independent component analyses (ICAs).

To obtain a comprehensive and objective understanding of this intrinsic activity and connectivity, a fast and data‐driven graph theory approach, FCD mapping, was proposed for large‐scale exploratory whole‐brain network analyses (Barkhof, Haller, & Rombouts, 2014; Tomasi & Volkow, 2010). The FCD mapping can be divided into short‐range and long‐range FCDs, based on the neighboring relationships between brain voxels, for which the spatial differentiating scale is usually between 10 and 15 mm (Beucke et al., 2013; Sepulcre et al., 2010; Zhang et al., 2015). Short‐range FCD (sFCD) at a voxel (x_0_) represents the number and strength of the voxels in the local FC cluster. sFCD might reflect local or regional functional interactions near x_0_, whereas long‐range FCD (lFCD) allows the identification of remote or long‐distance dense connections (functional hubs) with high sensitivity. sFCD and lFCD are distinct but also related. Previous studies have demonstrated that the functional property of local connectivity can affect remote connectivity, including whole‐brain dynamics (Deco et al., 2014). In the current study, we hypothesis that insufficient sleep during chronic insomnia could widely affect intrinsic functional activities and connectivity in network level, both local and remote connectivities. For that, FCD mapping was applied to investigate changes in cortical and subcortical functional connectivity in patients with CID during daytime resting states. First, both sFCD and lFCD maps were compared between patients with CID and healthy controls with good sleep groups (HGSs) to investigate the effect of insomnia on local or remote connectivity profiles. Next, we further investigated the connectivity networks due to the altered lFCD in such CID patients. In addition, a partial correlation analysis was conducted within the CID group to evaluate the relationships among clinical data, sFCD and lFCD values, and the iFC coefficients of the identified abnormal regions. Our results revealed abnormalities in both sFCD and lFCD between the brains of CID patients and HGSs.

## 2 MATERIALS AND METHODS

This case–control study was approved by the Medical Research Ethics Committee and the Institutional Review Board (No. 2,012,003) of the First Affiliated Hospital of Nanchang University. All subjects received written informed consent and were identified only by number before undergoing an MRI scan. All of the research procedures were performed according to the ethical principles of the Declaration of Helsinki and approved guidelines.

### 2.1 Subjects

From May 2012 to July 2013, 32 consecutive treatment‐naïve patients with CID (CID group) who were scheduled for neuropsychological examination, functional MRI scans, and clinical intervention were recruited from a university hospital. All insomnia patients had reported having difficulty initiating or maintaining sleep or having nonrestorative sleep with resulting daytime dysfunction or distress. These symptoms due to sleep insufficiency were not attributable to another medical or psychiatric disorder. For the CID group, the inclusion criteria were as follows: (1) age between 25 and 65 years with an independent psychiatric syndrome (primary insomnia); (2) a diagnosis according to the Diagnostic and Statistical Manual of Mental Disorders, version IV; and (3) a duration of symptoms of insomnia ≥ 1 year with sleep difficulty occurring at least 3 nights per week. The exclusion criteria were as follows: (1) any secondary sleep disorder (e.g., restless leg syndrome, obstructive sleep apnea) or sleep–wake rhythms disorders; (2) systemic diseases such as hypertension, heart disease, respiratory diseases, or diabetes; (3) a history or imaging evidence of cerebrovascular disease, head injury, or other neurological (epilepsy, neurodegeneration) or psychiatric (psychosis, current depression) diseases; (4) illicit drug or alcohol abuse or current intake of psychoactive medications; and (5) MRI contraindications, such as metallic implants, claustrophobia, or devices in the body, or subjects who did not finish the MRI scans or neuropsychological tests.

Thirty‐two well‐matched HGSs were recruited from the local community through advertisements. The HGSs reported having good sleep and were screened according to the exclusion criteria.

To ensure the quality of the rs‐fMRI data, each subject was limited to a maximum displacement in any cardinal direction (x, y, z) of less than 2 mm and a maximum rotation (x, y, z) of less than 2° (see the section on image preprocessing below).

### 2.2 Clinical evaluation of sleep quality and mood status

Each subject was assessed with a detailed clinical interview, a physical examination, and a clinical follow‐up, and all patients were self‐declared as being right‐handed and having normal vision. The sleep quality was assessed using Pittsburgh Sleep Quality Index (PSQI). Depression and anxiety were assessed using the Beck Depression Inventory‐II (BDI‐II), the State Trait Anxiety Inventory‐state (STAI‐s), and State Trait Anxiety Inventory‐trait (STAI‐t) before MRI scan.

### 2.3 MRI data acquisition

All participants were scanned on a 3.0 Tesla MRI system (Trio, Siemens) at the First Affiliated Hospital of Nanchang University. All of the subjects were instructed to avoid alcohol, caffeine, central nervous system‐active agents, or any other psychoactive substances for 48 hr prior to the MRI scan. Echo planar imaging (EPI) sequence was used for the re‐fMRI scanning with the following parameters: repetition time/echo time = 2000 ms/30 ms, matrix = 64 × 64, field of view = 200 mm × 200 mm, 30 interleaved axial slices, slice thickness = 4 mm, and slice gap = 1.2 mm. Each participant was required to lie quietly in the scanner with their eyes closed and was instructed not to think systematically or fall asleep during resting‐state scan (lasted for 480 s). For registration purposes, 3‐dimensional high‐resolution T_1_‐weighted anatomic images were obtained using a magnetization‐prepared rapid acquisition gradient‐echo sequence with the following parameters: repetition time/echo time = 1900 ms/2.26 ms, matrix = 240 × 256, field of view = 215 mm × 230 mm, and 176 sagittal slices with 1.0‐mm slice thickness and no gap. Additional conventional T_2_‐weighted and T_2_‐FLAIR (fluid‐attenuated inversion recovery) images were acquired in the brain to diagnose and exclusively diagnose. After scanning, the Epworth Sleepiness Scale (ESS) questionnaire was used to inspecting if the participant had fallen asleep during the scan.

### 2.4 Image preprocessing

Preprocessing of the rs‐fMRI data was performed by a toolbox for Data Processing & Analysis for Brain Imaging (Yan, Wang, Zuo, & Zang, 2016) (DPABI v2.1, http://rfmri.org/dpabi) based on the MATLAB (MathWorks, Inc.) platform. The first 10 time points for each subject were discarded to eliminate magnetic saturation effects. Slice timing and head motion corrections were performed sequentially for the remaining 230 time points. Paired subjects were excluded if the head movement of one individual was greater than 2° of angular rotation along any axis and greater than 2 mm of translation along any axis during functional MRI scanning. Similarly, we also evaluated differences in head motion between the CID and HGS groups according to the criteria of Jenkinson, Bannister, Brady, & Smith (2002) (see Table 1). For precise spatial normalization of the fMRI data, individual high‐resolution T1‐anatomic images were registered to the mean fMRI data, and the resulting aligned T1‐weighted images were segmented and transformed into standard Montreal Neurological Institute (MNI) space using the DARTEL toolbox (Goto et al., 2013). Furthermore, a group template was generated for fMRI processing and statistics. Motion‐corrected functional images were specially normalized to the group template using the transfer parameter estimated by DARTEL segmentation and resampled to 3 × 3 × 3 mm^3^ voxels. Subsequently, temporal band‐pass filtering (0.01–0.1 Hz) and detrending were applied to reduce low‐frequency drift and physiological high‐frequency respiratory and cardiac noise. Finally, white matter and cerebral–spinal fluid signals and 24 head realignment parameter (Friston model (Friston, Williams, Howard, Frackowiak, & Turner, 1996)) were regressed out as covariates.

**Table 1:** Demographic and clinical data of the subjects

	CID	HGS	*t*‐values (*p*)
Age (y)	42.59 ± 11.59	40.92 ± 11.46	0.531 (.598)
Gender (F/M)	17/10	17/10	0.000 (.999)
Education (y)	9.52 ± 3.04	10.51 ± 4.17	−1.006 (.3.19)
Duration of symptoms of insomnia (y)	10.98 ± 8.85	N/A	N/A
PSQI	13.29 ± 2.54	0.86 ± 1.06	23.489 (<.0001)
STAI‐s	28.07 ± 4.16	26.22 ± 8.14	0.531 (.298)
STAI‐t	32.25 ± 4.81	29.07 ± 10.01	1.479 (.145)
BDI‐II	6.15 ± 5.29	4.92 ± 1.59	01.148 (.256)
Mean head motion^a^	0.097 ± 0.066	0.098 ± 0.041	−0.030 (.977)

Note:

Data are presented as means ± standard deviations;.

Abbreviations: BDI‐II, Beck Depression Inventory‐II; CID, chronic insomnia disorder; HGS, healthy controls with good sleep; N/A, not applicable; PSQI, Pittsburgh Sleep Quality Index; STAI‐s, State Trait Anxiety Inventory‐state; STAI‐t, State Trait Anxiety Inventory‐trait.

^a^ Head motions were evaluated according to the frame‐wise displacement (FD) criteria described by Jenkinson et al. (2002).

### 2.5 Functional connectivity density mapping and statistical analysis

Based on the preprocessed rs‐fMRI data, sFCD and lFCD were calculated in each voxel of whole‐brain regions using GRETNA (Graph‐theoretical Network Analysis Toolkit: http://www.nitrc.org/projects/gretna) (Wang et al., 2015), according pervious study (Tomasi & Volkow, 2010). The FCD of each voxel in the binary brain network was calculated as the number of links or edges connected to a given voxel beyond the threshold of the correlation factor (classical reference: $r_{0}$ = .25(Buckner et al., 2009; Han et al., 2015; Zhou et al., 2015; Zuo et al., 2012); for review, see (Zuo et al., 2012)). Moreover, the cutoff points of sFCD and lFCD for short‐ and long‐range distances were set as 12 mm of anatomic distance according to previous studies (Beucke et al., 2013; Zhang et al., 2015). sFCD was calculated by counting the number of neighboring voxels within the neighborhood (radius sphere ≤ 12 mm) of directly surrounding brain regions for each voxel; lFCD was computed by the same procedure, except that only voxels outside the neighborhood were considered as contrasted with distant (radius sphere > 12 mm) interactions (Beucke et al., 2013; Sepulcre et al., 2010; Zhang et al., 2015). Pairs of voxels with Pearson's correlations (*r*) > .25 (corresponding to *p* < .001 for each connection in one‐sample *t* tests) were considered functionally connected in this study. sFCD and lFCD were standardized separately by conversion to Z scores to improve normality. Finally, all of the FCD results were spatially smoothed with a 6‐mm full width at half‐maximum (FWHM) Gaussian kernel and normalized to the average strength in the whole brain.

General linear model (GLM) and independent sample analysis was used to investigate group differences in the sFCD or lFCD between the CID and HGS groups, within the gray matter mask from high‐resolution T_1_‐weighted image after controlling for the effects of gender, age, and the mean frame‐wise displacement (FD). For multiple comparisons correction, the significance of voxel‐wise intensity was determined at *p* < .001, and the cluster‐level threshold was estimated by adaptation of Doug Ward's AlphaSim program within RESTplus V1.2 (http://restfmri.net/forum/RESTplusV1.2; parameters: rmm = 5, FWHM = 6 mm, and iterations = 1,000). Next, the FCD values (k) were extracted for each region showing significant group differences in the sFCD or lFCD. sFCDs and lFCDs were normalized separately using the averaged whole‐brain FCD values (k/k0) for Cohen's d post hoc analyses of the effect size (Parker & Hagan‐Burke, 2007). To determine whether the sFCD and lFCD results depended on the selection of the *r*_0_ value threshold, other correlation thresholds (e.g., *r*_0_ = 0.1, 0.15, 0.2, 0.3, 0.35, and 0.4) were used to recompute the sFCD and lFCDs maps. The resulting sFCD and lFCD maps were then used to perform the statistical analyses. Finally, a partial correlation analysis was also performed in the patients with CID to assess the relationships between the clinical data and the average normalized FCD measured in each region with altered sFCD or lFCD, with age and gender as covariates. We corrected the resulting p‐values using Benjamini–Hochberg false discovery rate (FDR)‐based multiple comparisons correction (*q*‐value < .05).

### 2.6 Connectivity patterns of the regions with altered lFCD and comparative analysis

To show the influence on the connectivity network, the mean time course of each seed region was extracted, and computed Pearson's correlation coefficient between the seed region and each voxel in the whole brain. The correlation coefficient values were normalized with Fisher's r‐to‐z transformation for group‐level analyses. Subsequently, a one‐sample *t* test in SPM12 was used to identify the significantly connectivity regions with each seed region for CID and HGS groups (*p* < .001, multiple comparisons correction with a two‐tailed false discovery rate (FDR)). We clarified that we adopted this method for multiple comparisons in one‐sample *t* test based on previous studies (Wang et al., 2014) and that it could provide a robust connectivity network for backtracking the disconnection of the regions with altered lFCD. While FDR controlling is strict for whole‐brain functional analysis, its procedures are designed to control the number of false discoveries (*type I errors*) rather than test all of the hypotheses, such as the family‐wise error rate (AlphaSim in this study) correlation. In this study, only positive correlations were considered a valid mask of the connectivity pattern in the one‐sample *t* test analysis, although this topic remains controversial (Meyer‐Lindenberg, Domes, Kirsch, & Heinrichs, 2011; Raichle, 2010; Wang et al., 2014; Yeo et al., 2011).

Next, an independent sample analysis was used to evaluate the group differences between the CID and HGS groups within the gray matter mask in SPM12, age, gender, and the mean FD values as covariate. The threshold was AlphaSim‐corrected (*p* < .001) using the RESTplus V1.2. In addition, Cohen's d analysis was performed in each region with significant group differences in iFC to conduct a post hoc analysis of the effect size. Finally, a partial correlation analysis was used to assess the clinical relationships of average iFC values in each region with altered iFC in the CID group, with age and gender as covariates. We also corrected the resulting p‐values using Benjamini–Hochberg FDR‐based multiple comparisons correction (*q*‐value < 0.05).

### 2.7 Statistical analyses of demographic and clinical data

All demographic and clinical data were statistically analyzed using SPSS version 13.0 (SPSS).

## 3 RESULTS

### 3.1 Demographics and clinical data of the participants

Ultimately, 27 treatment‐naïve patients with CID and 27 well‐matched HGSs were enrolled in this study. The demographic and clinical data of all participants are shown in Table 1. As expected, the patients with CID exhibited significantly higher PSQI scores than the HGSs (*t* = 23.979, *p* < .001), whereas no significant differences were found for the Beck Depression Inventory‐II (*t* = 0.970, *p* = .339), the STAI‐s (*t* = 0.539, *p* = .592), or the STAI‐t (*t* = 1.078, *p* = .286). No significant differences were found between the CID and HGS groups with regard to age, gender, years of education, or head motion (Jenkinson et al., 2002).

### 3.2 Spatial distribution of the sFCD and lFCD maps

**Figure 1:**
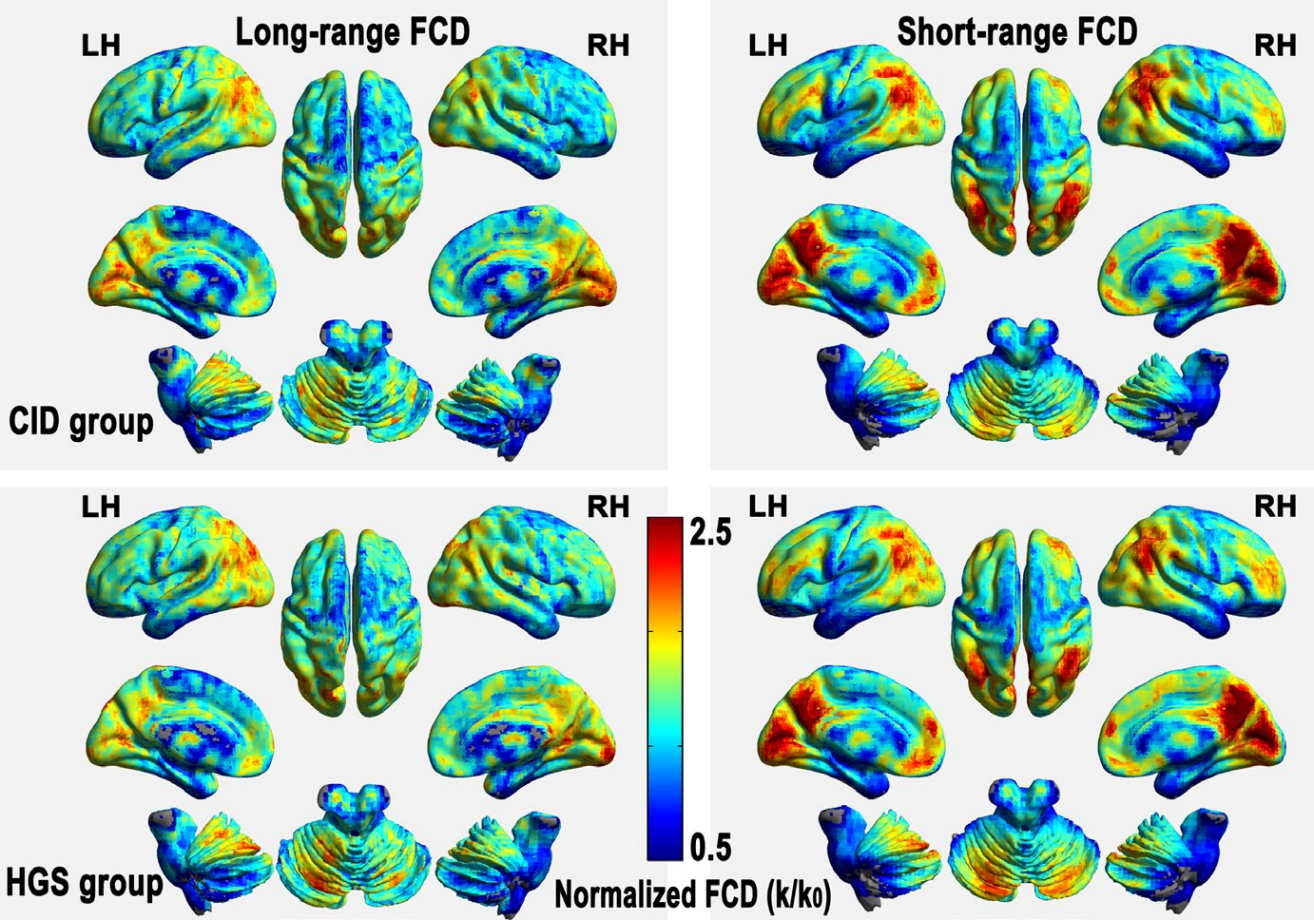
The spatial distributions of sFCD and lFCD in patients with CID and HGSs visualized using the Brainnet Viewer (http://www.nitrc.org/projects/bnv/). FCD, functional connectivity density; sFCD, short‐range FCD; lFCD, long‐range FCD; LH, left hemisphere; RH, right hemisphere; CID, chronic insomnia disorder; HGSs, healthy controls with good sleep groups

In this study, the sFCD and lFCD spatial distributions were identified in the CID and HGS groups using the respective group mean FCD values in Pearson's correlations ((*r*_0_) > .25) (Figure 1). The CID and HGS groups showed similar distributions of their relatively high sFCD regions, including the bilateral posterior cingulate cortices, precuneus, posterior parietal cortices, and occipital and dorsolateral prefrontal cortices (DLPFCs). The relatively high lFCD regions were also bilaterally distributed in both groups, including the cuneus, precuneus, posterior cingulate cortices, middle temporal gyrus (MTG), pre3.central gyrus, and DLPFCs. Similar patterns (upon visual inspection) of sFCD spatial distributions, IFCD spatial distributions, and between‐group differences at different Pearson's correlation thresholds (i.e., *r*_0_ = .1, .15, .2, .3, .35, and .4) are shown in Figure S1 and Figure S2.

### 3.3 Disrupted sFCD and lFCD in patients with CID

**Figure 2:**
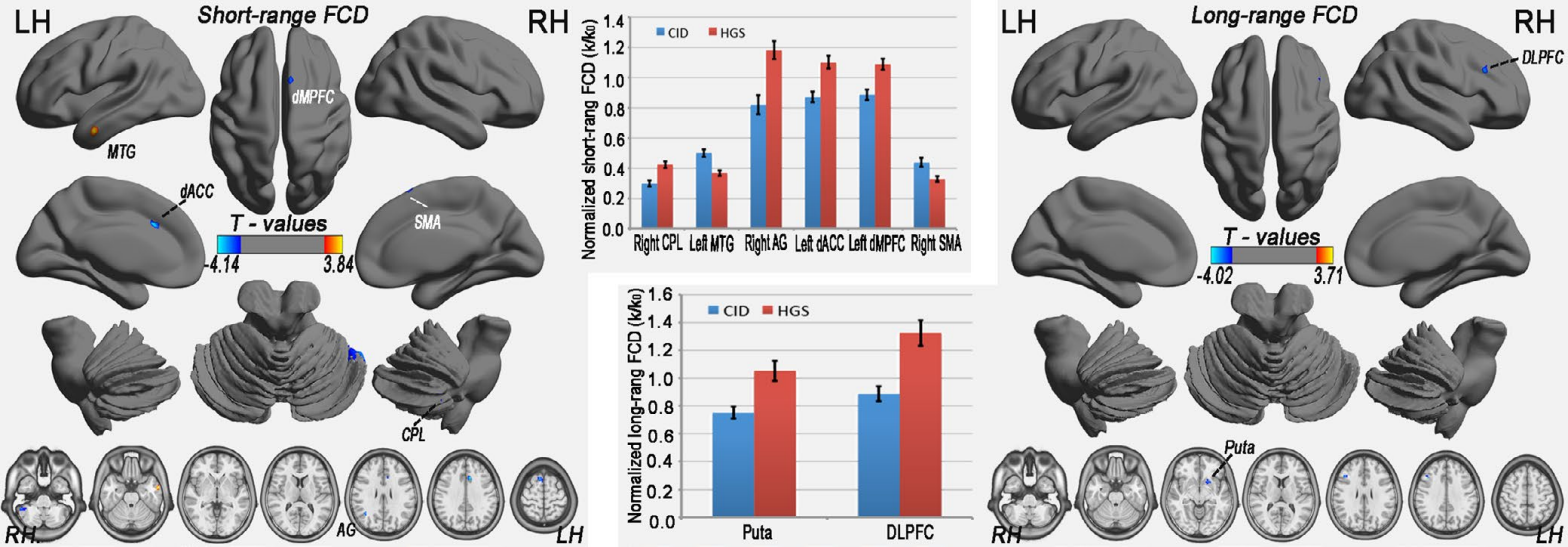
Brain regions with significant changes in sFCD (left) and lFCD (left) in patients with CID (3dClustSim‐corrected at *p* < .001) according to a two‐sample *t* test visualized using the Brainnet Viewer (http://www.nitrc.org/projects/bnv/) and the DPABI Viewer (http://rfmri.org/dpabi). Significant group differences in the mean strengths of lFCD (middle upper) and sFCD (middle bottom) were identified in the above regions (*p* < .001). LH, left hemisphere; RH, right hemisphere; FCD, functional connectivity density; sFCD, short‐range FCD; lFCD, long‐range FCD; dMPFC, dorsal medial prefrontal cortex; MTG, middle temporal gyrus; dACC, dorsal anterior cingulate cortex; SMA, supplementary motor area; CPL, cerebellum posterior lobe; AG, angular gyrus; DLPFC, dorsolateral prefrontal cortices; CID, chronic insomnia disorder; HGS, healthy controls with good sleep

In this study, we also report that the between‐group differences were conservatively restricted to connections with correlation coefficients above 0.25 (corresponding to *p* < .001 for each connection in one‐sample *t* tests), a classic reference *r*_0_ value. Compared with the HGSs, the patients with CID primarily exhibited decreased lFCD in the right DLPFC and left putamen (3dClustSim‐corrected at *p* < .001; Figure 2 and Table 2). Regarding sFCD, reductions were observed in the executive control system (left dorsal ACC, dACC), supplementary motor area (right SMA), basal ganglia (left putamen), and right cerebellum posterior lobe (CPL), whereas increased sFCD values were observed in the temporal regions (left MTG) and anterior DMN (left dMPFC) (3dClustSim‐corrected at *p* < .001; Figure 2 and Table 2). These regions all showed significant alterations (*p* < .001) with regard to the mean normalized FCD in the CID group, with a relatively large effect size (Figure 2).

**Table 2:** Brain regions showing differences in the sFCD and lFCD between the CID and HGS groups (3dClustSim‐corrected at *p* < .001)

Brain regions	BA	Cluster size (voxels)	MNI coordinates of peak (x, y, z)	Peak T‐scores	Effect size
Short‐range FCD (CID versus. HGS)					
Right cerebellum posterior lobe (CPL)		19	51, −48, −45	−3.280	1.261
Left middle temporal gyrus (MTG)	20,21	28	−57, −3, −27	3.835	1.253
Right angular gyrus (AG)	39,22	18	39, −57, 24	−3.684	1.167
Left dorsal anterior cingulate cortex (dACC)	32	15	−9, 21, 33	−4.137	1.176
Left dorsal medial prefrontal cortex (dMPFC)	6	10	−18, −6, 78	3.449	0.889
Right supplementary motor area (SMA)	6	26	9, 18, 63	−3.513	1.030
Long‐range FCD (CID versus. HGS)					
Left putamen (Puta)		29	−24, 15, −6	−3.631	0.974
Right dorsolateral prefrontal cortex (DLPFC)	48	19	42, 24, 30	−4.021	1.503

Abbreviations: BA, Brodmann's area; CID, chronic insomnia disorder; FCD, functional connectivity density; HGS, healthy controls with good sleep; lFCD, long‐range FCD; MNI, Montreal Neurological Institute; sFCD, short‐range FCD.

### 3.4 Relationships between clinical metrics and sFCD or lFCD

Among the patients with CID, partial correlation analyses between clinical metrics and sFCD and lFCD in brain regions showing significant group differences revealed the following: (1) decreased lFCD in the left putamen (FDR adjusted *p*‐value (*q*‐value) = 0.0009) and the right DLPFC (*q*‐value = 0.002) was significantly negatively correlated with the duration of symptoms of insomnia (Table 3); and (2) significant correlations were observed between the PSQI score and the sFCD in the right CPL (*q*‐value = 0.002), the left MTG (*q*‐value = 0.001), the right AG (*q*‐value = 0.002), the left dACC (*q*‐value = 0.002), the right dMPFC (*q*‐value = 0.011), or the right SMA (*q*‐value = 0.0009). However, no significant relationships were detected between other metrics (BD, STAI‐s and STAI‐t) and sFCD or lFCD (Table 3).

**Table 3:** Clinical metrics associated with the FCD in the CID patients (ρ/*P*/q values)

	Duration of symptoms of insomnia	STAI‐s	STAI‐t	BD	PSQI score
Normalized lFCD (k/k0) in the left putamen	−0.511/0.011*/0.165	−0.239/0.057/1.365	−0.268 /0.057/0.855	−0.214/0.132/1.980	−0.500/0.00018***/0.0009^###^
Normalized lFCD (k/k0) in the right DLPFC	−0.046/0.832/1.783	0.121/0.397/1.863	0.121/0.397/1.191	0.094/0.512/1.280	−0.448 /0.00097***/0.002^##^
Normalized sFCD (k/k0) in the right CPL	−0.200/0.348/1.740	−0.172/0.228/1.200	−0.172/0.228/1.710	−0.197/0.167/0.835	−0.472/0.00047***/0.002^##^
Normalized sFCD (k/k0) in the left MTG	0.081/0.706/1.765	0.066/0.647/1.617	−0.069/0.629/1.570	0.125/0.382/1.146	0.516 /0.0001***/0.001^##^
Normalized sFCD (k/k0) in the right AG	0.125/0.561/1.683	−0.127/0.375/1.406	−0.144/0.315/1.575	0.019/0.893/1.674	−0.482/0.0003***/0.002^##^
Normalized sFCD (k/k0) in the left dACC	0.176/0.412/1.545	0.197/0.166/0.830	0.130/0.363/1.361	−0.161/0.258/0.967	−0.455/0.0008***/0.002^##^
Normalized sFCD (k/k0) in the right dMPFC	0.412/0.045/0.337	−0.006/0.969/1.817	0.030/0.832/1.560	−0.033/0.818/1.753	0.381/0.0058**/0.011^#^
Normalized sFCD (k/k0) in the right SMA	−0.021/0.924/1.732	−0.021/0.884/1.894	−0.063/0.659/1.412	−0.205/0.149/1.117	−0.510/0.0001***/0.0009^###^

Abbreviations: AG, angular gyrus; BD, Beck Depression; CID, chronic insomnia disorder; CPL, cerebellum posterior lobe; dACC, dorsal anterior cingulate cortex; DLPFC, dorsolateral prefrontal cortices; dMPFC, dorsal medial prefrontal cortex; FCD, functional connectivity density; lFCD, long‐range FCD; MTG, middle temporal gyrus; PSQI, Pittsburgh Sleep Quality Index; sFCD, short‐range FCD; SMA, supplementary motor area; STAI‐s, State Trait Anxiety Inventory‐state; STAI‐t, State Trait Anxiety Inventory‐trait.

*p < .05, **p < .01, ***p < .001; #, ## and ###Significant with false discovery rate (FDR) correction for multiple comparisons at q = 0.05, 0.01, and 0.001.

### 3.5 The connectivity pattern of altered lFCD

**Figure 3:**
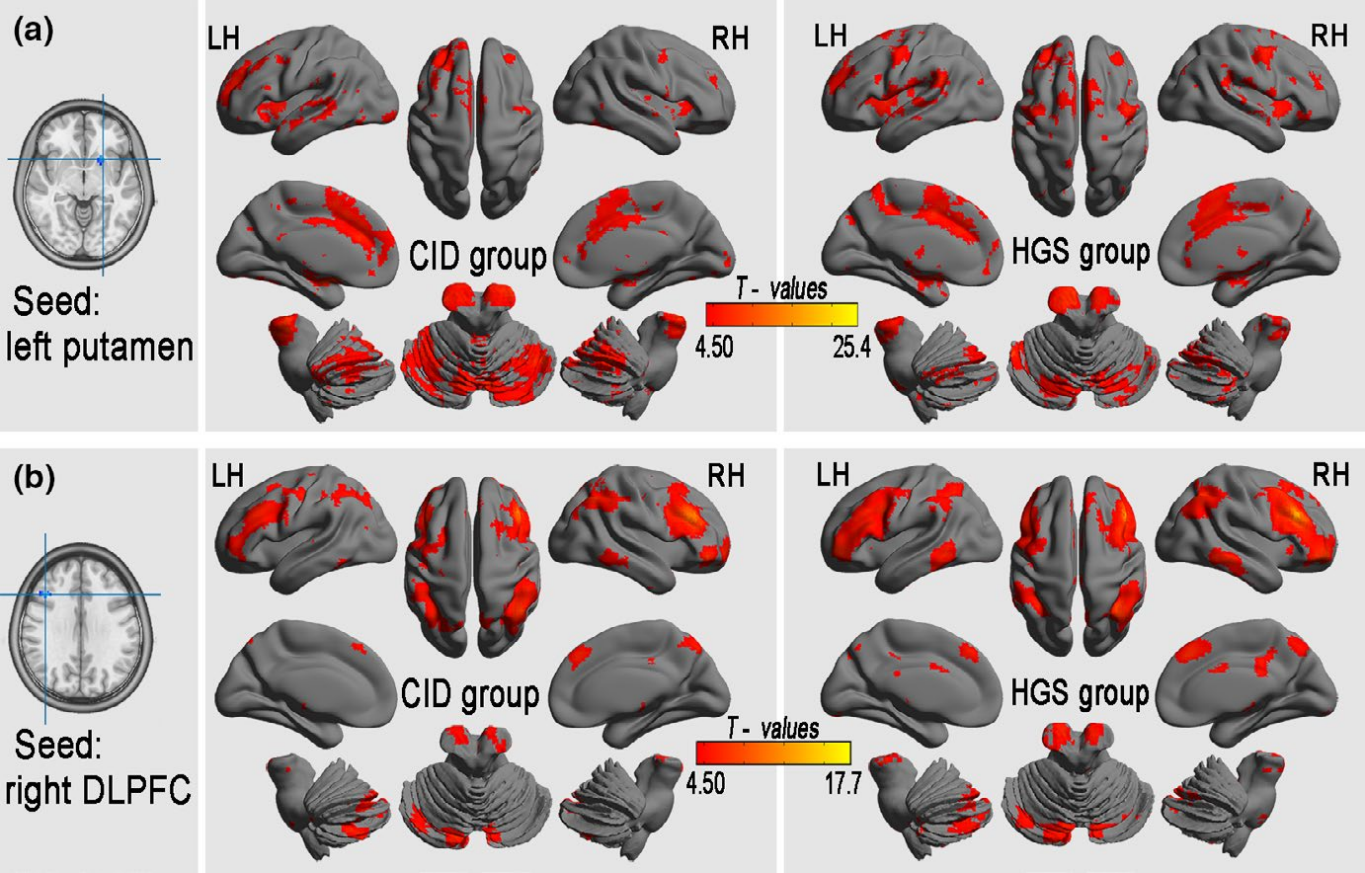
Connectivity patterns of the regions with altered lFCD in patients with CID and HGSs (*p* < .001, FDR‐corrected). lFCD, long‐range functional connectivity density; LH, left hemisphere; RH, right hemisphere; CID, chronic insomnia disorder; HGSs, healthy controls with good sleep groups; DLPFC, dorsolateral prefrontal cortices

**Figure 4:**
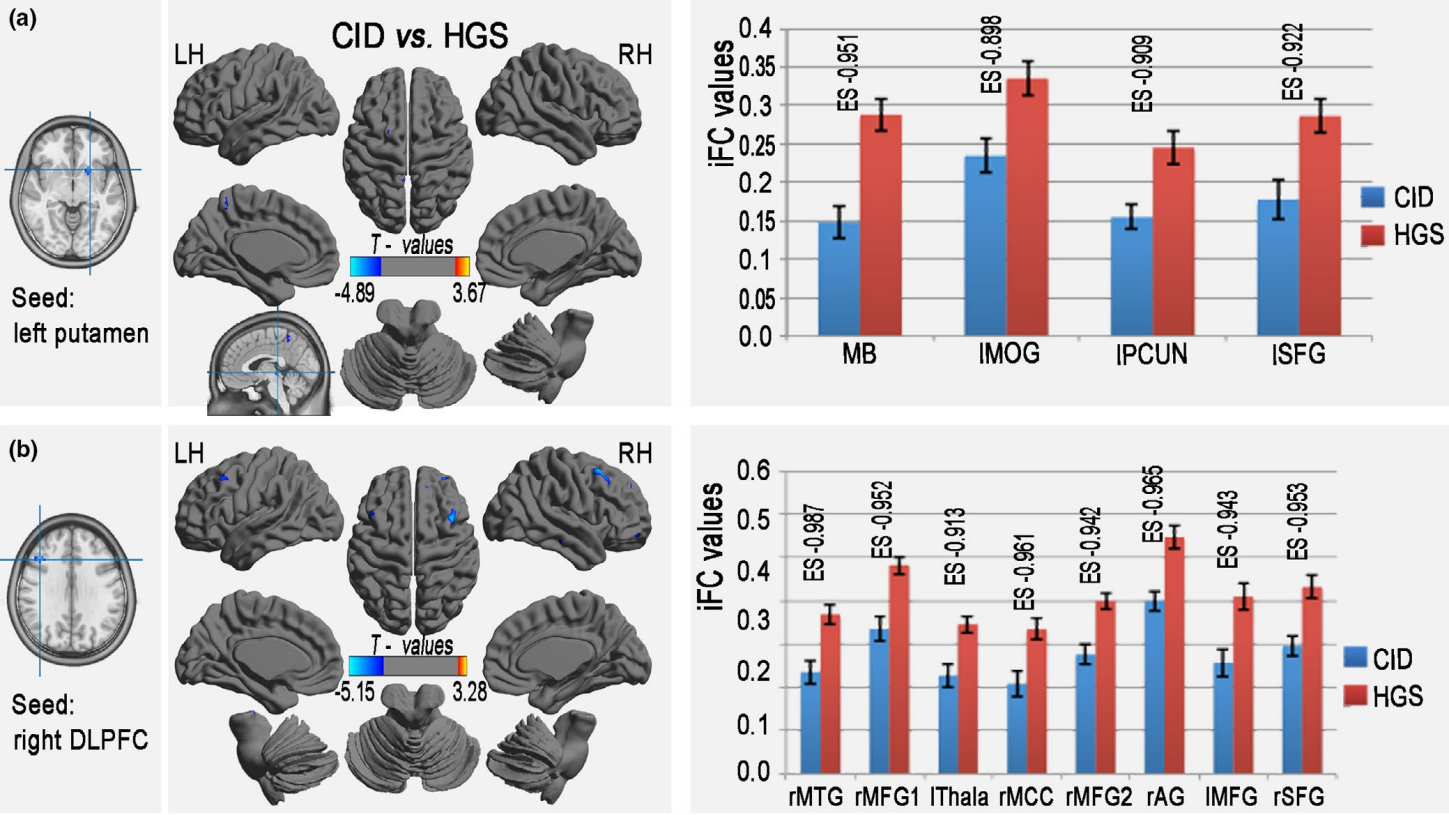
Group iFC differences in regions with altered lFCD (e.g., a：left putamen; b: right DLPFC) between the CID and HGS groups. The left and middle columns show the seed regions and the statistical significance (color‐coded T‐values) of the iFC patterns for each seed region with significant group differences in lFCD. The right column represents the effect size of brain regions with significant differences (3dClustSim‐corrected at *p* < .001) for all seed regions between the CID and HGS groups. iFC, intrinsic functional connectivity; lFCD, long‐range functional connectivity density; CID, chronic insomnia disorder; HGS, healthy controls with good sleep; DLPFC, dorsolateral prefrontal cortices; lMOG, left middle occipital gyrus; lPCUN, left precuneus; lSFG, left superior Frontal gyrus; rMTG, right middle temporal gyrus; rMFG, right middle frontal gyrus; rMCC, right middle cingulate cortex; rAG, right angular gyrus; lThala, left thalamus; lMFG, left middle frontal gyrus; rSFG, right superior frontal gyrus; LH, left hemisphere; RH, right hemisphere

One‐sample *t* tests were used to backtrack the connectivity patterns of all seed regions with altered lFCD. The results showed significant positive iFC regions (FDR‐corrected *p* < .001, corresponding to a threshold correlation coefficient *r*_0_ = 0.25 in the FCD statistics) in both the CID and HGS groups (Figure 3). A voxel‐based GLM analysis detected significantly reduced iFC in seed regions from areas with significant group differences in lFCD in the CID group compared with that in the HGS group (3dClustSim‐corrected at *p* < .001; Table 4, Figure 4). Specifically, the left putamen showed decreased iFC in the brainstem, left STG, right Rolandic operculum, right IPL, left superior frontal gyrus (SFG), right PrCG, left PCUN, right and left SMAs, and right and left SFG. The right DLPFC showed decreased iFC in the right MTG, left thalamus, right middle CG, right and left middle frontal gyrus (MFG), right angular gyrus, and right SFG. The right dMPFC showed decreased iFC in the left and right CPLs, left putamen, right BG, right PCUN, left thalamus, right MFG, and right SFG.

**Table 4:** Significant differences in the iFC in seed regions of the lFCD between the CID and the HGS groups (*p* < .001, AlphaSim corrected)

Brain regions	BA	Cluster size (voxels)	MNI coordinates of peak (x, y, z)	Peak T‐scores		Effect size
Seed from left putamen (CID versus. HGS)						
Midbrain		14	−3, −30, −3	−4.895		−0.951
Left middle occipital gyrus	19,39	14	−39, −81, 24	−4.178		−0.898
Left precuneus	7	41	3, −54, 60	−3.142		−0.909
Left superior frontal gyrus	6	11	−21, 3, 72	−3.226		−0.922
Seed from right DLPFC (CID versus. HGS)						
Right middle temporal gyrus	20,21	33	69, −24, −21	−3.280		−0.987
Right middle frontal gyrus 1	10	122	33, 54, −9	−4.542		−0.952
Left thalamus		12	−6, −6, 12	−3.440		−0.913
Right middle cingulate gyrus	23	22	9, −33, 33	−3.226		−0.961
Right middle frontal gyrus 2	9,8,6	94	39, 15, 54	−5.157		−0.942
Right angular gyrus (AG)	40	21	57, −57, 48	−3.370		−0.965
Left middle frontal gyrus	8	67	−45, 21, 48	−4.036		−0.943
Right superior frontal gyrus	8	12	15, 51, 39	−4.092		−0.953

Abbreviations: CID, chronic insomnia disorder; DLPFC, dorsolateral prefrontal cortices; HGS, healthy controls with good sleep; iFC, intrinsic functional connectivity; lFCD, long‐range FCD; MNI, Montreal Neurological Institute.

### 3.6 Relationships between the clinical metrics and iFC values of altered lFCD

**Figure 5:**
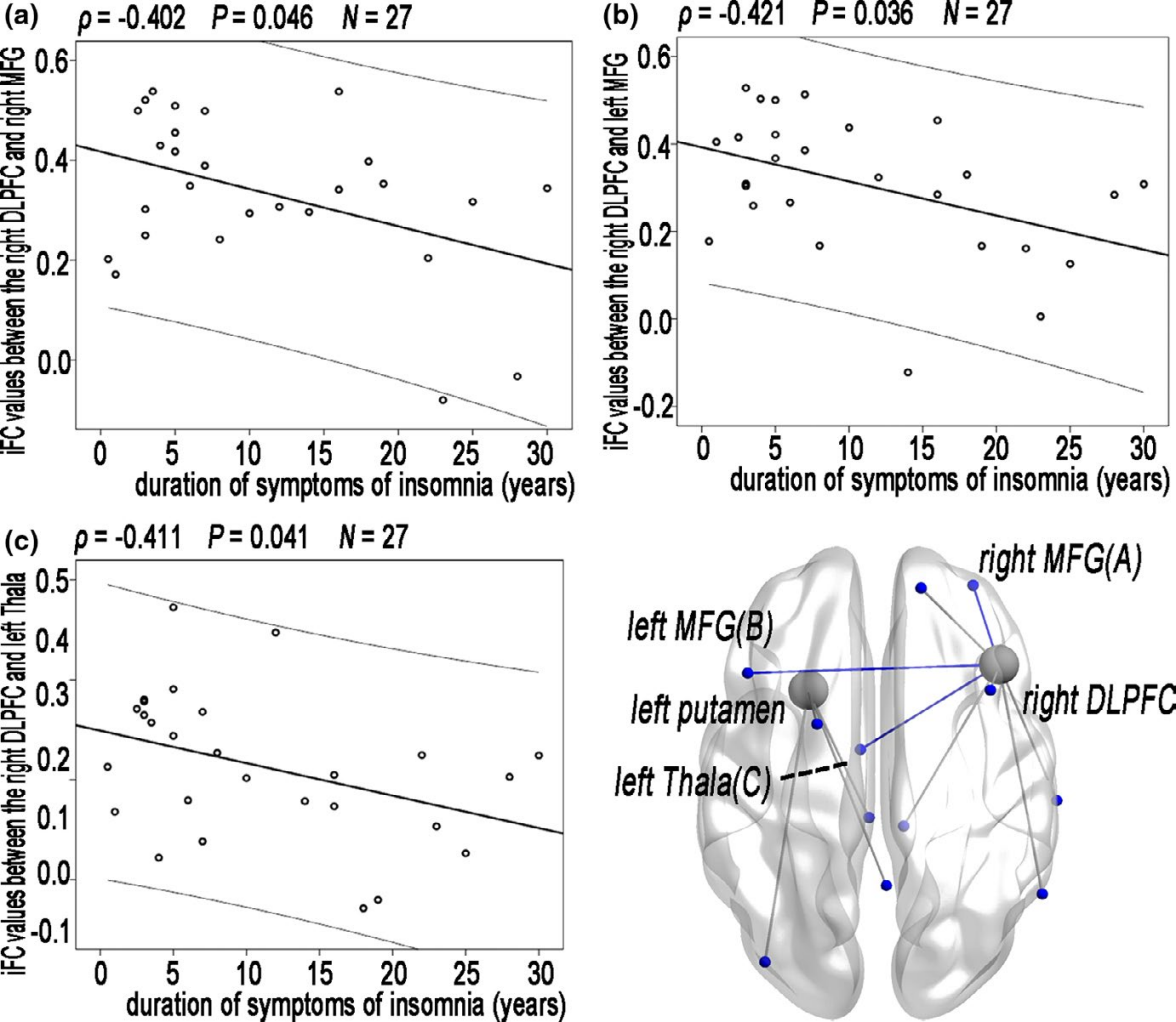
Decreased iFC values in regions with altered lFCD associated with sleep quality or the duration of symptoms of insomnia in patients with CID; however, this correlation did not remain after FDR correction. iFC, intrinsic functional connectivity; lFCD, long‐range functional connectivity density; CID, chronic insomnia disorder; FDR, false discovery rate; DLPFC, dorsolateral prefrontal cortices; MFG, middle frontal gyrus; Thala, thalamus

Partial correlation analyses among the patients with CID revealed that a trend toward correlation between the clinical metrics and iFC values of altered lFCD (Figure 5); however, this correlation did not remain after FDR correction (Table S1).

## 4 DISCUSSION

This study used sFCD and lFCD mapping to investigate alterations in the strength of inter‐ and intraregional iFC in patients with CID at rest. Our study primarily found that patients with CID showed significantly decreased lFCD in the right dMPFC and left putamen, reflecting diminished intrinsic activity, impaired interregional communication (phase delay) or a reduced number of connections. Furthermore, the patients with CID showed decreased sFCD in the left dACC, right SMA, right AG, and right CPL, as well as increased sFCD in the left MTG and left dMPFC, reflecting a regional reduction in local connectivity and hyper‐homogeneity of local activity in the somatosensory association cortex. In addition, most regions in which patients with CID showed decreased lFCD also showed significantly decreased iFC in the frontal and temporal cortices within the same group.

### 4.1 Decreased sFCD in patients with CID

This study primarily observed that patients with CID showed significantly reduced sFCD in the left dACC, right SMA, right AG, and right CPL. These areas are involved in daytime behavior and in the functional system according to their neuroanatomical‐related function. Furthermore, we observed that the decreased sFCD in the above regions was correlated with PSQI score. The MOG belongs to the secondary visual cortex, and damage to the MOG is associated with visual agnosia(Renier et al., 2010). The dACC is a core component of the executive control system and is connected to the prefrontal and parietal cortices and the motor system; this area processes top‐down and bottom‐up stimuli and assigns appropriate control to other areas in the brain (Spunt, Lieberman, Cohen, & Eisenberger, 2012). Other decreased sFCD regions, including the right SMA, right AG, and right CPL, interact to control movement.

Impaired daytime function is the most common reason why people with insomnia seek help (Schutte‐Rodin, Broch, Buysse, Dorsey, & Sateia, 2008). Individuals commonly report symptoms of fatigue and irritability, as well as impaired concentration and performance in social and occupational domains. Previous structural MRI studies identified gray matter reductions in the hippocampus (Noh et al., 2012), ACC (Joo et al., 2013) and orbitofrontal cortex (Altena et al., 2008). Functional connectivity network studies conducted during the daytime have found evidence of dysfunction in cognitive performance (Drummond et al., 2013; Huang et al., 2012; Li et al., 2016b; Li et al., 2014). A ^18^F‐fludeoxyglucose (^18^F‐FDG) PET demonstrated that a smaller reduction in relative metabolism in wakefulness in the ascending reticular activating system, medial prefrontal cortex, and ACC (O'Byrne, Berman Rosa, Gouin, & Dang‐Vu, 2014). In particular, regional brain activity studies have observed decreased amplitudes of low‐frequency fluctuations (ALFFs) in the bilateral CPLs, left dMPFC, bilateral limbic lobe, and left occipital gyrus (Zhou, Huang, Zhuang, Gao, & Gong, 2016), as well as decreased ReHo in the right middle cingulate cortex and left fusiform (Wang et al., 2016). In this study, the reduced local FCD suggests that chronic insomnia is closely related to a disrupted local connectivity pattern with diminished intrinsic activity or phase delay as a possible neural cause of the functional loss or lower waking metabolism; however, whether this disrupted connectivity is associated with impaired daytime functioning requires further investigation in the future.

### 4.2 Increased sFCD in patients with CID

Consistent with previous studies of ALFFs (Zhou, Huang, Zhuang, et al., 2016) and ReHo (Wang et al., 2016), we detected increased sFCD in the temporal regions (left MTG) and anterior DMN (left dMPFC). The increased sFCD observed in the left MTG in this study is involved in processes of recognition (Ellison, Schindler, & Milner, 2004), the perception of facial emotions (Radua et al., 2010), and the reception of sensory inputs from the brain (Bjoertomt, Cowey, & Walsh, 2009). Neural activity in the dMPFC has been suggested to integrate information from distinct sensory areas. Previously, patients with CID were found to exhibit significantly increased intrinsic activity at rest, indicating that analogous excessive hyperarousal mechanisms exist during the daytime (Riemann et al., 2010). Hyperarousal processes play a key role in the pathophysiology of primary insomnia. Moreover, a recent study of sleep‐deprived participants showed increased (“compensatory”) task‐related activation and reduced deactivation in part of the DMN (Drummond et al., 2013). This reduced deactivation might indicate that insomniacs engage in a greater degree of externally oriented processing at even the lowest level of task difficulty. Thus, we demonstrated that a lack of sleep over long periods of time impairs the cerebral inhibitory control system in patients with CID at rest. In theory, this alteration may result in a hyperarousal state in certain regions with abnormal intrinsic sensory processing (Riemann et al., 2010).

### 4.3 Decreased lFCD in patients with CID

Decreased lFCD was observed in the right DLPFC and left putamen of the patients with CID. As a part of the central‐executive network (CEN), the DLPFC is connected to the orbitofrontal cortex and secondary association areas of the neocortex, including the posterior temporal, parietal, and occipital areas (Hoshi, 2006). In particular, the left and right DLPFCs exhibited reduced iFC in the right MTL, as well as in the right and left dMPFCs. The MTL and dMPFC are part of the DMN. Specifically, Yu et al. (2018) reported an imbalanced neural spontaneous fluctuation in DMN (increased anterior and decreased posterior DMN) in the resting state in CID patients. In addition, the decreased iFC associated with disrupted lFCD in the DLPFC was related to the duration of symptoms of insomnia in this study. The reduced lFCD in the right DLPFC and the decreased iFC might reflect general disruption due to long‐term insufficient sleep; furthermore, this disruption might aggravate the effect on executive function, cognitive and emotional processing functions through a secondary decline lFCD in the right dMPFC. Combining these previous findings and our present results, we could speculate that the disrupted long‐range intrinsic connectivity might be responsible for lower execution.

A recent large GWAS study demonstrated that the genetic component of insomnia points toward a role of genes enriched in locomotory behavior, and enriched in the specific cell types from the medium spiny neurons (Jansen et al., 2019). The putamen is one of the major nuclei of medium spiny neurons (γ‐aminobutyric acid ergic inhibitory cells), connected to many other structures, and has many functions, including controlling motor skills. Furthermore, the putamen plays roles in many types of learning (Packard & Knowlton, 2002). Disrupted structural connections (Spiegelhalder et al., 2014) and the reduced local amplitude of the functional activity (Zhou, Huang, Zhuang, et al., 2016) of the putamen might support the disconnection of the putamen in patients with CID. In addition, we found a stronger negative correlation between the PSQI score and normalized lFCD of the left putamen or right DLPFC, indicating that disconnection of the left putamen was associated with poor sleep quality responses to long‐term insomnia among patients with CID.

This study did not find increased regions of lFCD, which is different from previous studies of gFCD (Yu et al., 2018), possibly due to the stage of the CID patient.

In addition, both the sFCD and lFCD results reflect unilateral regional differences, such as the left putamen and right DLPFC for lFCD outcomes. Why does chronic insomnia only affect a single unilateral brain area and not the corresponding bilateral functional networks? This question should be answered by targeting the laterality of insomnia in future investigations.

Finally, this study focused on alterations in sFCD and lFCD, and FCD was computed as the number of edges between voxel *x*_0_ and other connected voxels with an arbitrary correlation threshold (a decreased false‐positive rate). In a previous study, Yan, Yang, Colcombe, Zuo, & Milham (2017) demonstrated that different rs‐fMRI indices (i.e., iFC, fractional ALFF, ReHo, or other indices) exhibit similar patterns of variation across time; that is, network connectivity strengths were selectively correlated with ALFFs (Tal et al., 2013). Nevertheless, these rs‐fMRI indices were not designed to replace existing metrics. Our results in this study are partially consistent with the findings of previous iFC, ALFF, ReHo, or other rs‐fMRI studies, such as disrupted local connectivity (ReHo) in emotion‐related regions and sensorimotor regions (Wang et al., 2016) or decreased ALFFs in several DMN subregions and the prefrontal cortex (Zhou, Huang, Gao, et al., 2016). However, these findings are not fully consistent with each other. Therefore, our findings for sFCD and lFCD, together with those of other CID investigators (Chen et al., 2014; Huang et al., 2012; Wang et al., 2016), can be used to guide the selection of individual indices and can facilitate comparison and integration of findings across studies using different indices in future studies.

This study has several technical and biological limitations that must be acknowledged. First, the acquired images were of a limited spatial resolution due to the 3‐mm slice thickness; moreover, the cutoff points for the short‐ and long‐range distances (12 mm) limited the spatial resolution, and the cutoff points may affect the results according to previous studies (Beucke et al., 2013; Zhang et al., 2015). Future studies should utilize higher‐resolution fMRI and more cutoff points for distances. Second, this study had a small sample size, and the neuropsychological correlations of both sFCD and lFCD should be interpreted with caution because we did not objectively assess sleep quality, daytime sleepiness, or the level of alertness in the scanning session. In addition, the ESS was used to assess the level of alertness at the end of the scanning session; however, this questionnaire is purely subjective, and drowsiness or simply falling asleep in the scanner might overlay the effects of drowsiness and sleep stage on resting‐state functional connectivity. In the future, a more reliable method to rule out sleepiness should also be used. In the end, for the different methods for multiple comparisons, we clarified that 3dClustSim‐correction was used to provide a cluster‐extent‐based threshold approach to test the hypotheses of the group comparison in this study. The aim of the use of different multiple comparison correction methods was to adjust the type I error rate (FDR correction) to discriminate real differences between the observed data without compromising statistical power. We also provide the results with the same criteria for multiple comparisons (in one‐ and two‐sample *t* test; Figure S3) and without multiple comparisons (Figure S4) in the supporting information for reference, and the results of the between‐group differences were exactly similar among the different criteria. Indeed, it is quite difficult to say which is better or more correct among the multiple comparison correction methods.

## 5 CONCLUSIONS

The current study obtained a specific new finding in brain lFCD and sFCD in patients with CID using FCD mapping. Insufficient sleep in patients with CID may widely affect cortical functional activities or phase delay, including decreased lFCD distributed in the dorsolateral prefrontal cortices, key regions of the CEN and putamen and decreased sFCD distributed in the anterior DMN, executive control network, and supplementary motor‐related areas. Furthermore, several regions showed increased sFCD in the patients with CID. Our findings might improve our understanding of the comprehensive dysfunction in patients with CID. Furthermore, lFCD and sFCD should be considered when exploring iFC‐related clinical manifestations.

## CONFLICTS OF INTEREST

The authors declare that there are no conflicts of financial or nonfinancial interest regarding the publication of this article.

## AUTHOR CONTRIBUTIONS

F.Z., X.Z., and H.G. formulated, conceived, and designed the research. S.H., M.H., and J.J. collected some of the MRI data samples. F.Z., Y.Z., and L.H. preprocessed and analyzed the data. F.Z. and Y.Z. contributed reagents/materials/analysis tools. F.Z., Y.Z., M.H., J.J., S.H., and H.G. wrote the manuscript.

### Peer Review

The peer review history for this article is available at https://publons.com/publon/10.1002/brb3.1844.

## Supporting information

Supplementary Material

## Data Availability

The datasets generated during the current study are available from the corresponding author upon reasonable request (fq.chou@yahoo.com).
