# Supplementary material for: Altered long‐ and short‐range functional connectivity density associated with poor sleep quality in patients with chronic insomnia disorder: A resting‐state fMRI study
Source: Brain Behav. 2020 Sep 16;10(11):e01844. doi: 10.1002/brb3.1844 (PMC7667361; doi:10.1002/brb3.1844)
Supplement: Supplementary file 1 — Supplementary Material [file BRB3-10-e01844-s001.docx]

**Supporting Information**

**
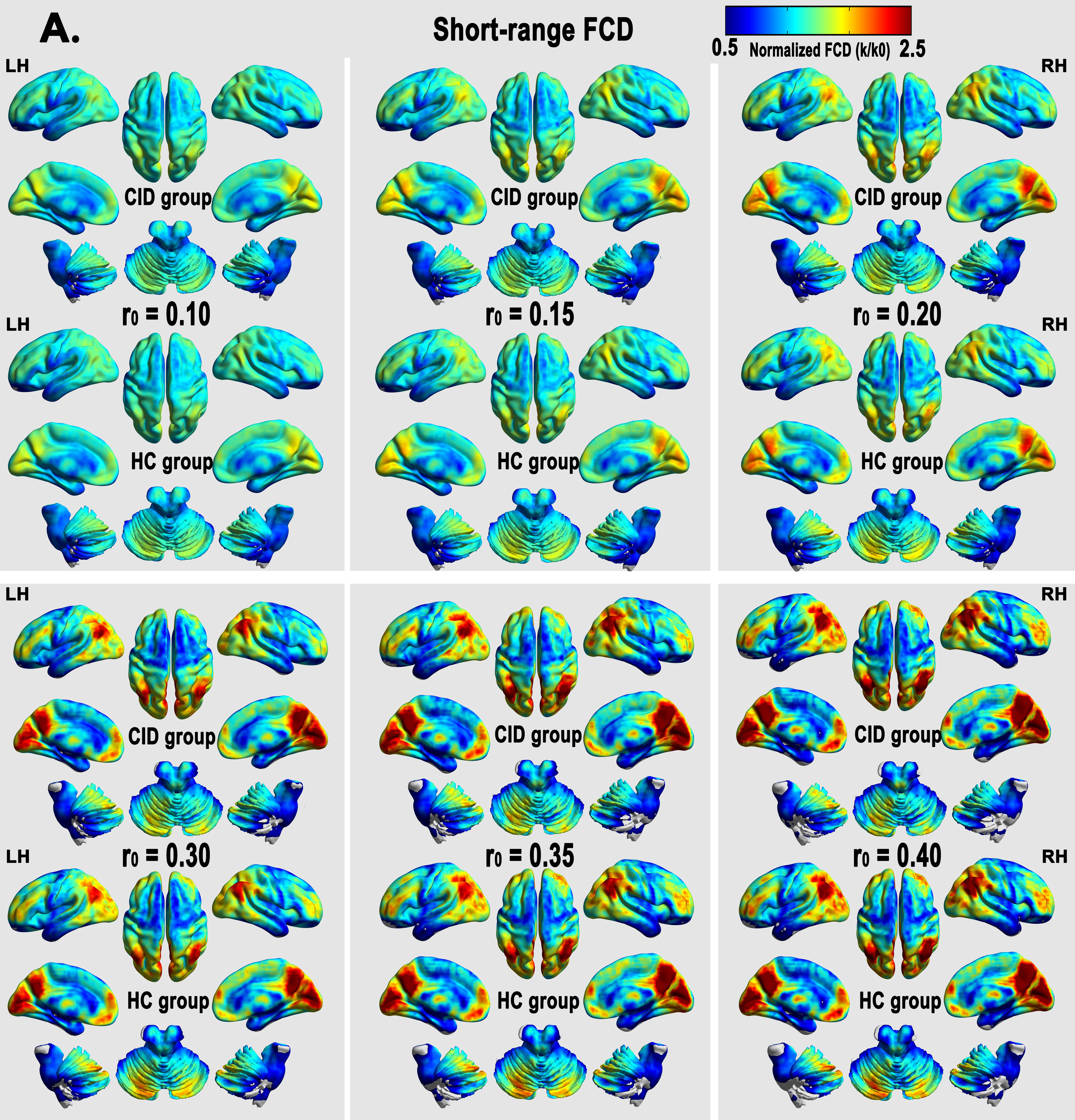

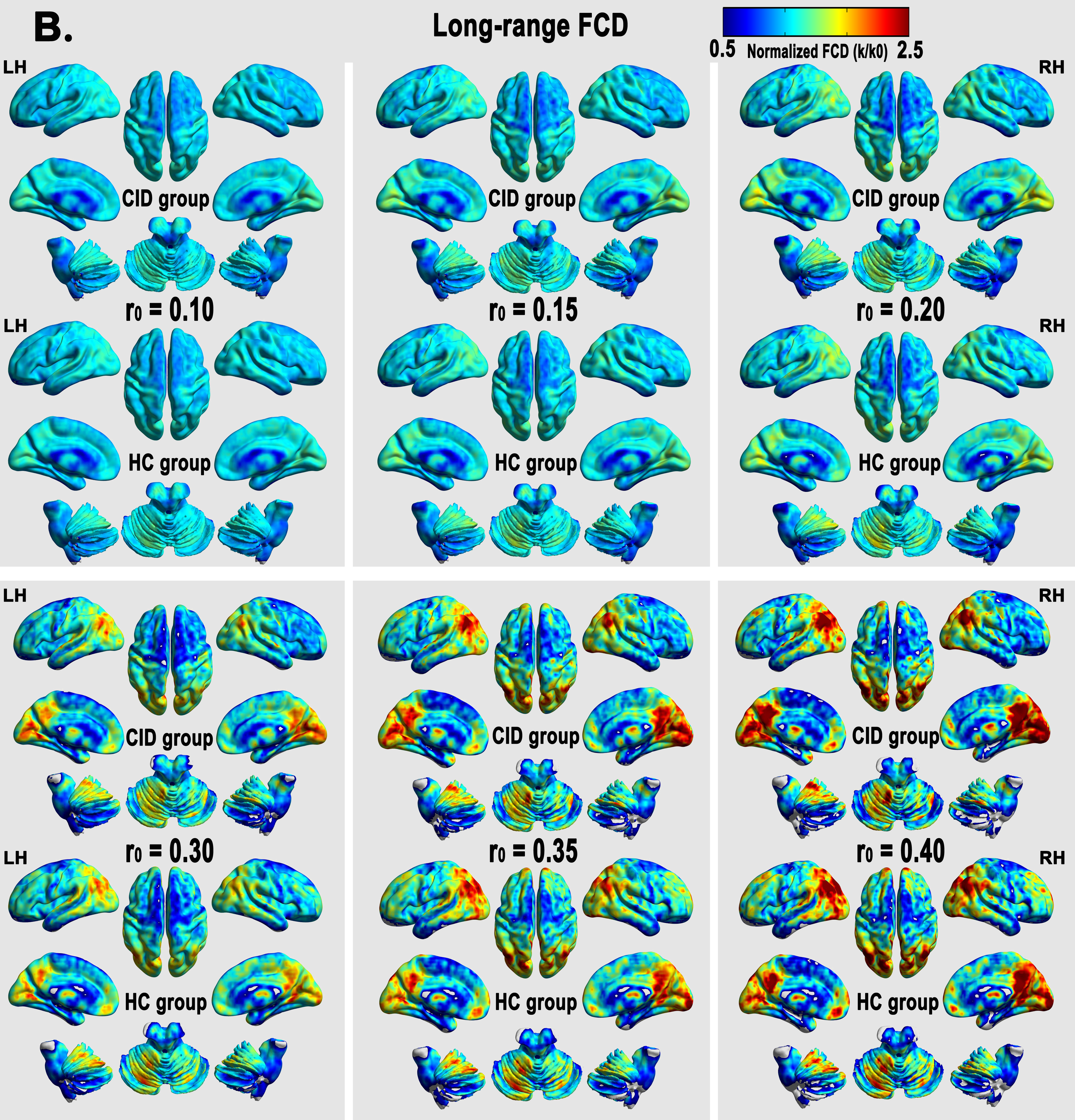
**

**Figure S1** The sFCD (A) and lFCD (B) spatial distribution maps for the different correlation thresholds (e.g., $r_{0}$ = 0.1, $r_{0}$ = 0.15, $r_{0}$ = 0.2, $r_{0}$= 0.3, $r_{0}$ = 0.35, and $r_{0}$ = 0.4). (TIF)

**
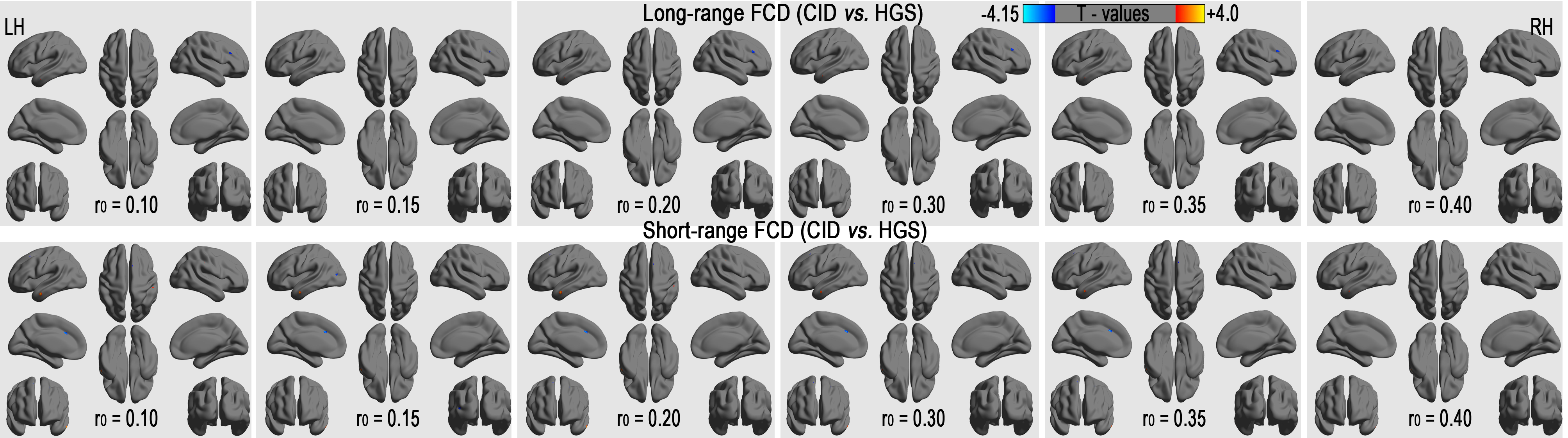
**

**Figure S2** Between-group differences in the FCS in patients with CID compared using the different cut-off correlation thresholds (e.g., $r_{0}$ = 0.1, $r_{0}$ = 0.15, $r_{0}$ = 0.2, $r_{0}$= 0.3, $r_{0}$ = 0.35, and $r_{0}$ = 0.4), P < 0.01, with 3dClustSim-corrected. (TIF)

**
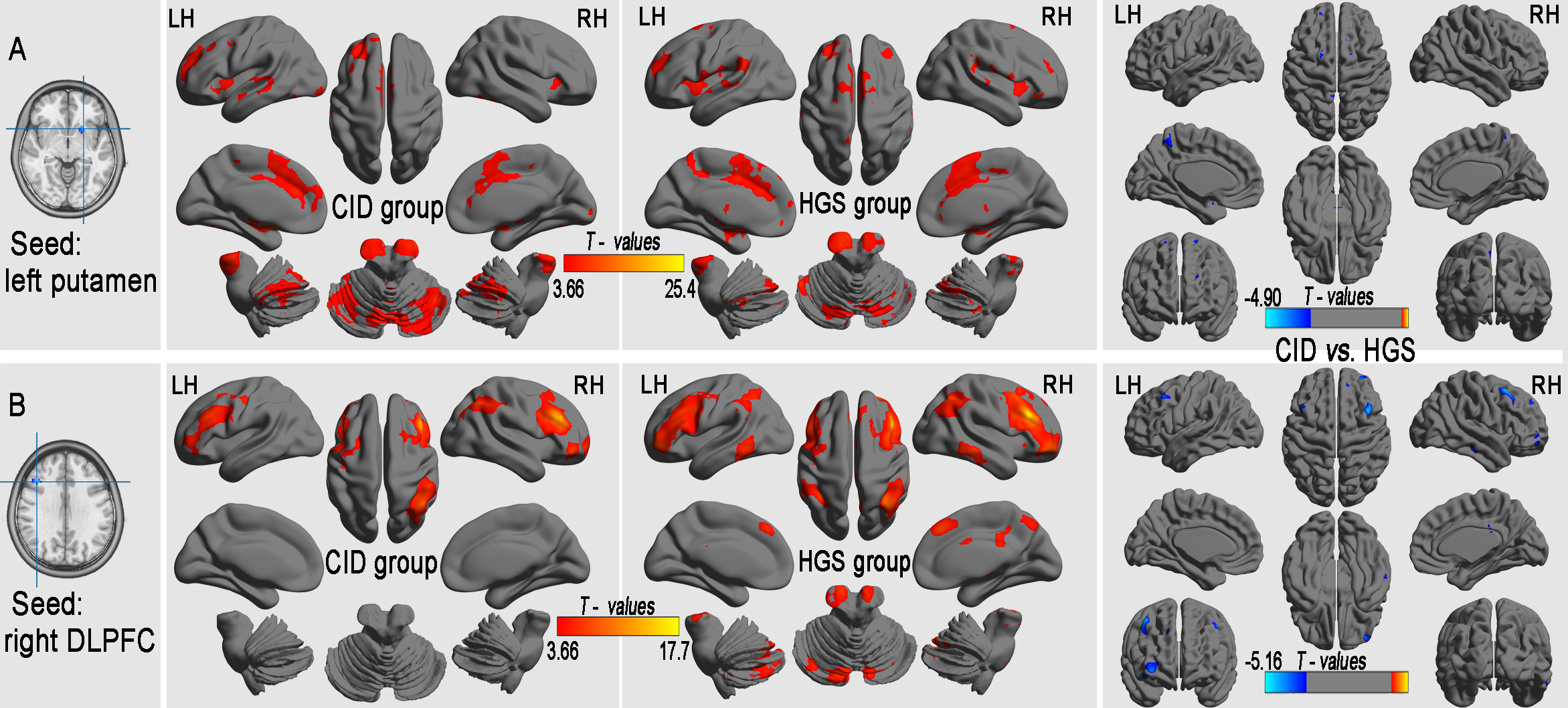
**

**Figure S3** Connectivity patterns (middle column) and between-group differences (right column) of the regions with altered lFCD in patients with CID and HGSs (P < 0.001, 3dClustSim-corrected, respective). (TIF)

**
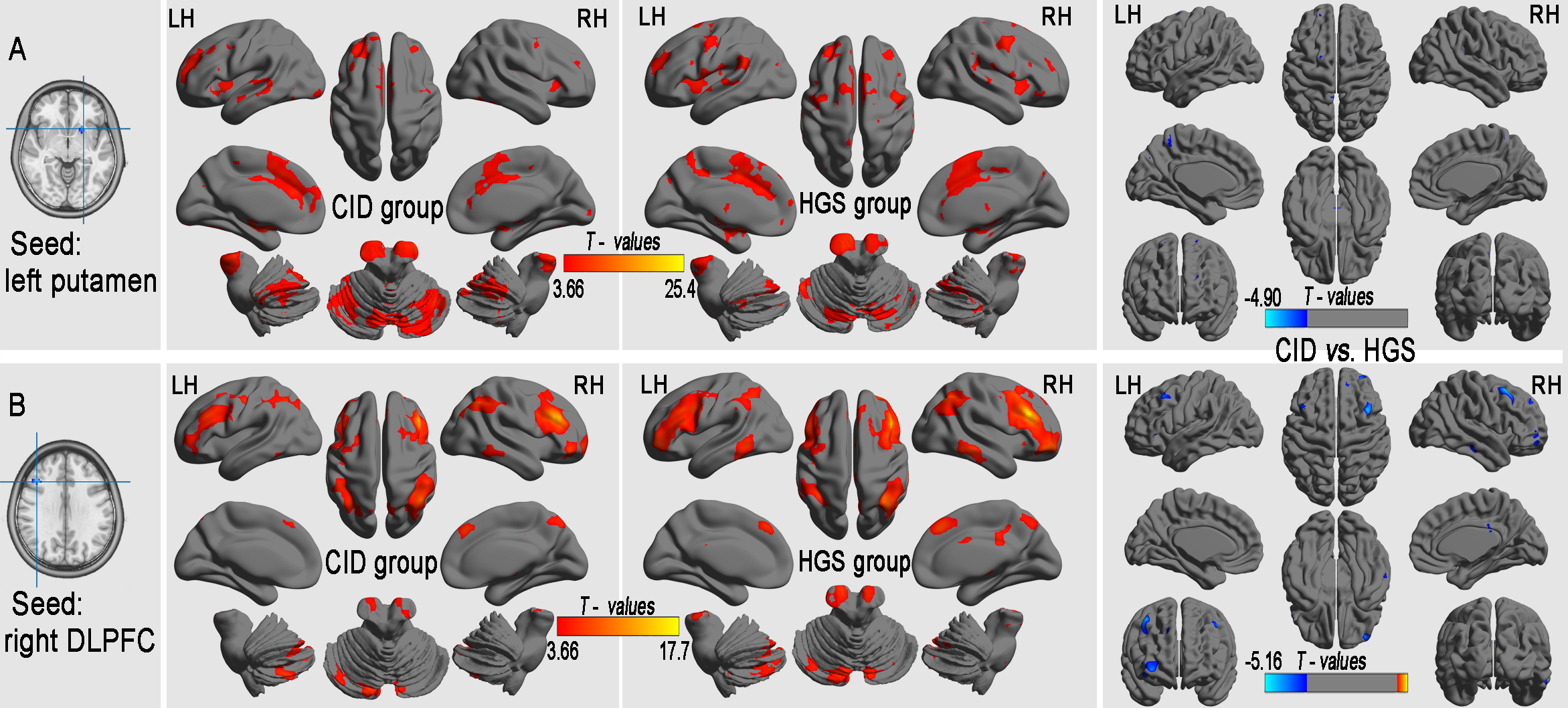
**

**Figure S4** Connectivity patterns (middle column) and between-group differences (right column) of the regions with altered lFCD in patients with CID and HGSs (P < 0.001, without correction, respective). (TIF)

**Table S1** Clinical metrics associated with the iFC in the CID patients (ρ/P/q values).

|  | Age (years) | Duration of insomnia | STAI-s | STAI-t | BD-II | PSQI score |
| --- | --- | --- | --- | --- | --- | --- |
| rsFC values between the left putamen and midbrain | -0.049/0.815/1.222 | -0.178/0.395/0.658 | -0.120/0.569/2.133 | -0.088/0.675/1.265 | -0.093/0.658/0.822 | -0.135/0.520/1.300 |
| rsFC values between the left putamen and left MOG | -0.042/0.842/1.148 | -0.343/0.094/0.282 | 0.061/0.771/0.889 | -0.090/0.669/1.433 | -0.355/0.082/1.230 | -0.066/0.754/1.131 |
| rsFC values between the left putamen and left PCUN | 0.173/0.408/1.224 | 0.088/0.677/0.846 | 0.177/0.398/1.990 | 0.332/0.105/1.575 | 0.235/0.257/0.642 | 0.325/0.113/1.847 |
| rsFC values between the left putamen and left SFG | 0.216/0.300/1.125 | -0.089/0.674/0.919 | -0.230/0.268/2.010 | -0.196/0.347/1.735 | -0.290/0.159/1.192 | -0.047/0.824/1.123 |
| rsFC values between the right DLPFC and right MTG | -0.225/0.280/1.400 | -0.270/0.191/0.477 | -0.168/0.423/3.172 | -0.125/0.551/2.066 | -0.247/0.234/1.170 | -0.216/0.299/2.242 |
| rsFC values between the right DLPFC and right MFG 1 | -0.276/0.181/2.715 | -0.398/0.049*/0.245 | -0.061/0.774/1.451 | 0.006/0.978/1.630 | 0.264/0.203/1.522 | -0.110/0.600/1.000 |
| rsFC values between the right DLPFC and left Thala | -0.069/0.744/1.594 | -0.408/0.043*/0.322 | -0.021/0.920/1.380 | 0.005/0.982/1.473 | -0.214/0.305/0.915 | -0.193/0.354/1.770 |
| rsFC values between the right DLPFC and right MCC | -0.153/0.466/1.747 | -0.197/0.346/0.576 | 0.080/0.705/2.115 | 0.091/0.664/1.992 | -0.232/0.264/0.990 | -0.040/0.848/1.272 |
| rsFC values between the right DLPFC and right MFG 2 | 0.028/0.893/1.488 | -0.245/0.237/0.444 | -0.094/0.657/2.463 | -0.022/0.918/1.967 | 0.121/0.564/0.846 | -0.114/0.588/1.103 |
| rsFC values between the right DLPFC and right AG | 0.096/0.648/1.944 | -0.355/0.082/0.307 | -0.024/0.908/1.513 | 0.014/0.946/1.773 | -0.168/0.421/0.902 | -0.342/0.094/1.410 |
| rsFC values between the right DLPFC and left MFG | 0.089/0.672/1.680 | -0.192/0.357/0.535 | 0.098/0.640/3.200 | 0.172/0.410/3.075 | -0.339/0.097/1.455 | -0.149/0.477/1.431 |
| rsFC values between the right DLPFC and right SFG | -0.061/0.772/1.447 | -0.252/0.224/0.480 | 0.238/0.251/3.765 | 0.241/0.245/3.675 | 0.122/0.560/0.933 | -0.122/0.560/1.400 |

Note: * P < 0.05.
